# Stability and Dissolution Behavior Changes After Drug Compounding for Pediatric Cardiovascular Pharmacotherapy

**DOI:** 10.3390/pharmaceutics17040535

**Published:** 2025-04-19

**Authors:** Jumpei Saito, Akimasa Yamatani, Yuna Kojima, Masayoshi Nakakuni, Kosuke Nakano, Kaoru Hirose, Hidefumi Nakamura, Takehisa Hanawa, Miki Akabane

**Affiliations:** 1 Department of Pharmacy, National Center for Child Health and Development, 2-10-1 Okura, Setagaya-ku, Tokyo 157-8535, Japan; 2 Pediatric and Perinatal Medicine, Department of Clinical Pharmacy, School of Pharmaceutical Sciences, Meiji Pharmaceutical University, 2-522-1 Noshio, Kiyose-shi, Tokyo 204-0004, Japan; yamatani-a@ncchd.go.jp; 3 Department of Medical Design and Clinical Pharmaceutical Design, Faculty of Pharmaceutical Sciences, Tokyo University of Science, 2641 Yamazaki, Noda, Chiba 278-8510, Japant-hanawa@rs.tus.ac.jp (T.H.); 4 Department of Multicenter Collaboration, Network Promotion Unit, Clinical Research Center, National Center for Child Health and Development, 2-10-1 Okura, Setagaya-ku, Tokyo 157-8535, Japan; 5 Department of Research and Development Supervision, Clinical Research Center, National Center for Child Health and Development, 2-10-1 Okura, Setagaya-ku, Tokyo 157-8535, Japan

**Keywords:** pediatric compounding, cardiovascular drugs, ingredients stability, dissolution behavior

## Abstract

**Background:** Compounding is performed to adjust dosages and support medication for children. In Japan, tablets are crushed, diluted with lactose, and stored in bottles or sachets until use, but the stability and impact on dissolution of the ingredients after crushing have not been evaluated. **Methods:** Using a database established by the National Center for Child Health and Development in collaboration with 11 medical facilities, the status of tablet crushing was investigated. Commonly compounded drugs were selected as the target drugs. The selected drugs were sieved through a 500 μm mesh after crushing and diluted with lactose hydrate. The stability at 25 ± 2 °C/60 ± 5% relative humidity and the dissolution of the ingredients were evaluated after storing them for up to 120 days under the following conditions: (I) stored in a closed polycarbonate bottle (closed), (II) bottle opened once a day (in-use), or (III) stored in a laminated cellophane and polyethylene sachet (laminated). The changes in the ingredient content and dissolution behavior were evaluated in accordance with the Japanese Pharmacopoeia. **Results:** Five cardiovascular drugs (amlodipine besylate, carvedilol, propranolol hydrochloride, hydrochlorothiazide, and tadalafil) were selected as target drugs. No more than 10% change in ingredient content was observed for all five formulations compared to day 0. In addition, no related substances (impurities) were detected at more than 0.01%. There was no change in the dissolution rate of the samples after 120 days of storage under each storage condition. **Conclusions:** The five cardiovascular drugs commonly compounded for children in Japan maintained their pharmaceutical quality after compounding, even after long-term storage.

## 1. Introduction

In the absence of an adjustable dosage form or a dosage form suitable for children to take, the dosage form is modified by crushing tablets or opening capsules and preparing a powder formulation [1,2]. In these compounding processes in Japan, tablets are primarily crushed, or capsules are opened, and then a diluent, such as lactose, is added. The compounded powder formulation is then administered to children directly or treated as solutions after dissolving these compounded powders in water or syrups [1,2,3]. Currently, the stability of ingredients, physicochemical changes, and dissolution changes are not necessarily carried out after compounding in clinical settings. In Japan, powdered medicine is dispensed in single-dose packages (sachets) using a one-dose packaging machine. For this reason, it is necessary to ensure a certain amount of powder is dispensed, and lactose hydrate and starch are used as excipients (diluents) [1]. Pharmaceutical companies disclose information on the compounding of some drugs in response to requests from medical professionals, or the stability of the drug after compounding is presented in the drug information prepared by the pharmaceutical companies. However, the information disclosed by pharmaceutical companies does not include studies on the stability of the drug after dilution with lactose hydrate, reflecting the actual clinical practice in Japan. The stability of the drug in conditions involving repeated removal from a drug container or laminated packaging (packaged in sachets) remains to be studied. The effects on drug dissolution behavior in vehicles after compounding are also not yet understood. Despite the existence of pediatric indications and dosing information, some drugs do not have appropriate dosage forms for children. In clinical settings, when no suitable dosage form is available for children, compounding (crushing tablets or opening capsules) is conducted in the pharmacy [2].

Previously, we investigated the physicochemical stability and dissolution behavior of three ingredients—hydrocortisone, clonidine hydrochloride, and baclofen—after compounding [4,5,6,7]. These target ingredients were selected because they are frequently crushed and diluted with lactose hydrate at single and multiple facilities in Japan. The previous studies aimed to establish standardized compounding protocols to maintain the quality of in-house compounded preparations in Japan [4,5,6,7]. Powdered formulations that have been prepared as in-hospital preparations are stored in containers until the time of dispensing. When prescriptions are ordered by a physician and prepared for individual patients, they are dispensed in single doses and stored in hospital wards or at home in individual sachets. In particular, the stability of the product after crushing and diluting with an excipient such as lactose hydrate and the effect of crushing on the rate of dissolution are not evaluated by pharmaceutical companies. The product’s dissolution rate after compounding is also rarely investigated. In addressing the matter of “therapeutic orphans of pediatric formulation”, which pertains to the dearth of suitable pediatric formulations for children and caregivers in clinical settings, it is imperative to ensure the quality of formulations after compounding and storage under various conditions, such as containers or laminated sachets, that reflect the storage conditions prevalent in medical facilities and home care settings.

In the field of pediatric cardiological pharmacotherapy, numerous pharmaceutical products are frequently compounded for precise dosage adjustment and dispensed as powder medicines. According to a survey by the Pharmacy Department of the National Center for Child Health and Development, eight of the top ten drugs for neonates (aged up to one month old) and seven of the top ten drugs for infants (aged up to two years old) are cardiovascular drugs, and these drugs are frequently compounded from adult dosage forms for precise dosage adjustment [2]. However, there is a paucity of data regarding the quality of these compounded in-house preparations used in pediatric cardiological pharmacotherapy in actual clinical practice. The provision of quality-ensured in-house compounded preparations that guarantee the safety of drug therapy in children is imperative. The dissemination of information on standardized compounding methods may also contribute to maintaining the effectiveness and safety of cardiological pharmacotherapy for children, including neonates.

This study investigated the current trends in drug compounding in Japan. We examined the stability study after tablet crushing, dilution with lactose hydrate, and storage under certain conditions to establish the standardized compounding process in Japan.

## 2. Materials and Methods

### 2.1. Survey of Compounding Information

In order to obtain the latest information on compounding in children, we extracted information on compounding from the Pediatric Medical Information Collection System database (P-MICS database, https://pharma-net.ncchd.go.jp/en/database/information.html (accessed on 1 December 2024), which is maintained by the Pediatric and Drug Information Collection Network Project at the National Center for Child Health and Development, Japan. The period for prescription was set from 1 July 2019 to 30 June 2021. Regarding prescription data, including patients’ age, drug names, and dosage, permission to use the database was obtained from the National Center for Child Health and Development (approved on 24 October 2023, application number: 2023-126, data management representative; K.N.).

### 2.2. Selection of Products for Pharmaceutical Quality Evaluation

Among the products that are frequently crushed, cardiovascular drugs were chosen. The selection criteria included two key factors: firstly, a high number of cases of crushing, and secondly, drugs that are crushed at multiple facilities without bias. Subsequently, the quality of each drug post-processing (tablet crushing) was examined, encompassing the stability of drugs (including the measurement of related substances) after powdering and the addition of lactose or other excipients to obtain a certain drug concentration. Additionally, the existing information on dissolution behavior was reviewed. Finally, according to the above selection criteria, drugs frequently compounded in many medical facilities, for which no information is available after compounding, were selected.

### 2.3. Reagent and Test Solution Preparation for Stability Study

All reagents and solvents, including water, were of high-performance liquid chromatography (LC) analysis grade as standard substances for the target drugs; amlodipine, carvedilol, hydrochlorothiazide, propranolol, and tadalafil were purchased as standard substances with a purity of 98% or higher (Sigma-Aldrich, Tokyo, Japan). The following impurities were available as related impurities: Carvedilol impurity C ((2RS)-1[benzyl[2-(2-methoxyphenoxy)ethyl]amino]-3-(9H-carbazol-4-yloxy)propan-2-ol) (FUJIFILM Wako Pure Chemical Corporation, Tokyo, Japan) as an impurity of carvedilol, chlorothiazide (Tokyo Chemical Industry Co., Ltd., Tokyo, Japan) and salamide (Hydrochlorothiazide Impurity B, LGC Limited, Middlesex, UK) as an impurity of hydrochlorothiazide, and propranolol impurity-A ((2RS)-3-(Naphthalen-1-yloxy)propane-1,2-diol), impurity-B (1,1′-[(1-Methylethyl)imino]bis[3-(naphthalen-1-yloxy)propan-2-ol] Hydrochloride), and impurity-C (1,3-Bis(1-naphthalenyloxy)-2-propanol) as impurities of propranolol. These impurities were examined by comparing the spectra of the reference materials. For impurities that were difficult to obtain as reference materials, only the chromatograms of the following impurities were investigated: amlodipine impurities A, B, D, E, F, G, and H for impurities of amlodipine; propranolol impurities d, E, F, G, H, I, and J for impurities if propranolol; and tadalafil impurities A, B, C, D, and E for impurities of tadalafil. Lactose monohydrate (extra-fine crystalline lactose hydrate, Viatris Healthcare, Tokyo, Japan) was used as the diluent.

### 2.4. Compounding of the Target Drugs

Compounding each target drug was conducted by the pharmacist in the National Center for Child Health and Development dispensing room, in accordance with the pharmacy building and facilities regulations [8]. A total of 500 tablets of amlodipine besylate (Norvasc^®^ Tablets 10 mg, Viatris Healthcare LLC, Tokyo, Japan), 500 tablets of carvedilol (Artist^®^ Tablets 10 mg, Daiichi Sankyo Company, Limited, Tokyo, Japan), 1000 tablets of hydrochlorothiazide (Hydrochlorothiazide Tablets 12.5 mg “TOWA”, Towa Pharmaceutical Co., Ltd., Osaka, Japan), 500 tablets of propranolol (Inderal^®^ Tablets 10 mg, Taiyo Pharma Co., Ltd., Tokyo, Japan), and 120 tablets of tadalafil (Adcirca^®^ tablets, Nippon Shinyaku Co., Ltd., Kyoto, Japan) were each crushed in an automatic tablet crusher (KC-HUK2, Konishi Medical Instruments Co., Ltd., Osaka, Japan) at 6000 rpm for 30 s. The pharmaceutical products with the largest market share were selected as target products. The crushed tablets were filtered through a No. 30 test sieve (Tokyo Screen Co., Ltd., Tokyo, Japan) approved by the Japanese Pharmacopoeia. The sieved powder was mixed with lactose hydrate, and finally, amlodipine besylate, carvedilol, hydrochlorothiazide, propranolol, and tadalafil were diluted to 10 mg/g, 10 mg/g, 50 mg/g, 10 mg/g, and 20 mg/g, respectively (the final weight after dilution with lactose was 500 g for amlodipine besylate, carvedilol 500 g, hydrochlorothiazide 250 g, propranolol 500 g, and tadalafil 120 g). Compounded in-house preparations were obtained after mixing for 60 sec at 620 rpm of rotation and 20 rpm of revolution using an automatic mixer (YM-500, Yuyama Mfg. Co., Tokyo, Japan). The final concentration of each drug in the powder mixed with lactose was selected based on the real-world prescription data from the P-MICS database, with the dispensing amount of powder for a single dose ranging from 0.1 to 0.3 g.

### 2.5. Drug Stability Test

#### 2.5.1. Stability Test Conditions

The prepared powdered drugs were stored at 25 ± 2 °C/60 ± 5% relative humidity (RH) in a constant temperature and humidity chamber (Yamato Scientific Co., Ltd., Tokyo, Japan) [9]. The stability of the prepared target drugs after storage for a certain period of time was evaluated using samples taken on days 0, 30, 60, 90, and 120 after storage under the following three storage conditions [10]:

- Storage condition (1) “Bottle (closed)” test: Stored in amber polycarbonate bottles with desiccant without opening during storage periods.
- Storage condition (2) “Bottle (in-use)” test: 0.1 g of the powder was removed each day from the polycarbonate bottle to simulate the conditions of use in a real-world clinical setting.
- Storage condition (3) “Laminated” test: 0.3 g of the sample was pre-packaged in cellophane and polyethylene sachets (TK70W, Takazono Sangyo Co., Ltd., Tokyo, Japan).

For the quantitative evaluation of ingredients contents and impurity observation, 0.1 g of the sample was taken under each storage condition, diluted with a 50% (*v*/*v*) methanol aqueous solution, and then diluted with each mobile phase to the maximum concentration within the calibration curve range of the target compound to make the test solution.

#### 2.5.2. Detection Equipment

Detection was carried out using the LC-diode array detection (DAD) method that had been validated in prior studies, with reference to previous research reports [11,12,13,14,15,16]. The Ultimate 3000 LC System (Thermo Fisher Scientific K.K., Tokyo, Japan), which is equipped with an autosampler, column oven, and DAD, was used as an LC system. The autosampler was set at 10 °C and the column oven at 40 °C. For chromatographic separation, a C18 column (Imtakt US-C18 column, length 150 mm, inner diameter 3.0 mm, particle size 1.7 µm, Imtakt Co., Ltd., Kyoto, Japan) was used, and separation was carried out at flow rates ranging from 0.1 to 0.4 mL/min. The mobile phase used for the elution of each target ingredient and the separation mode were referred from previous studies [11,12,13,14,15,16]. The eluent was filtered through a 0.22 µm filter (Merck Millipore, Darmstadt, Germany), and 10 µL was injected into the LC-DAD. Data recording and analysis were performed using Chromeleon software version 6.80 (Thermo Fisher Scientific K.K., Tokyo, Japan).

#### 2.5.3. Standard Solutions, Calibration Curve, and Analytical Validation

The control solutions for quality assessment were prepared as 50% (*v*/*v*) methanol/water mixtures at concentrations of 20 (high-quality control, HQC), 2 (middle-quality control, MQC), and 0.2 (low-quality control, LQC) µg/mL for each target compound and known impurity and were prepared using standard materials. The test solutions for the calibration curve were prepared by dissolving 1.0 g of the stored powder (amlodipine 10.0 mg, carvedilol 10.0 mg, hydrochlorothiazide 50.0 mg, propranolol 10.0 mg, tadalafil 20.0 mg,) in 100 mL of a 50% (*v*/*v*) methanol/water mixture, and then serially diluted with a mixture of mobile phase solvents for high-performance LC to produce a seven-point concentration series (100, 50, 10, 5, 1, 0.5, and 0.1 μg/mL). Three independent dilution series were prepared and evaluated for intraday and interday variability. The criterion for acceptable linearity of the calibration curve was a correlation coefficient of 0.99 or greater. The lower limit of quantification (LLOQ), the lowest concentration of the calibration curve sample, was determined based on the ratio of signal (S) to noise (N). The quality control samples (HQC, MQC, and LQC) were measured three times a day on three different days. The data obtained were statistically analyzed using one-way analysis of variance, and the intra- and inter-day reproducibility was calculated in terms of accuracy (coefficient of variation, %CV) and precision (percentage of the detected concentration divided by the set concentration). The acceptable ranges for accuracy and precision were set at less than 15% and within 20%, respectively.

#### 2.5.4. Stability Evaluation

The stability of the target compounds of each drug was evaluated using the established quantitative methods. The main ingredients in the test samples were quantified after 30, 60, 90, and 120 days of storage in three different conditions (storage condition (1): bottle (closed), storage condition (2): bottle (in-use), storage condition (3): laminated), under light shielding, at 25 ± 2 °C/60 ± 5% RH. The acceptable range for the content of the target drug in each powder was set at 90.0–110.0% of the initial content after compounding on day 0 [17].

#### 2.5.5. Detection of Known or Unknown Impurities

Unknown or known impurities were identified using DAD. For known impurities that were available as reagents, information on the retention time of each impurity substance was collected using standard materials, and they were identified and quantified during the stability tests. The content of each impurity was evaluated by comparing the relative peak area of the main ingredient. The judgment method followed the International Council for Harmonization of Technical Requirements for Pharmaceuticals for Human Use (ICH) guidelines (ICH Topic Q 3 B (R2) Impurities in New Drug Products Step 5: Note for guidance on impurities in new drug products) [18]. The maximum dosage of the targeted drug after compounding for children differs depending on the case. The guidelines set the threshold for detection at 1.0% for maximum doses of 2 g or more, and 0.1% for doses of less than 1 mg. The threshold for quantifying impurities is set at 10 times this value. In this study, considering the maximum risk, the qualification threshold for quantifiable impurities was set at 1 μg (0.1% assuming a maximum dose of 1 mg). In addition, regardless of whether or not standard materials were available, 0.01% was set as the threshold for detection, and cases where 0.01% or more were judged to be non-compliant. In addition, detecting peaks for impurities and target compounds was judged to be non-detectable if the S/N ratio was less than 1.0.

### 2.6. Dissolution Test

The dissolution test was conducted in accordance with the paddle method of the Japanese Pharmacopoeia 17.6.10, which was harmonized by the ICH guidelines (NTR-6400AC, Toyama Sangyo, Tokyo) [19]. Either the First and Second fluid in the Japanese Pharmacopoeia dissolution test, diluted McIlvaine buffer solution (Fujifilm Wako Pure Chemical Corporation, Tokyo, Japan), or distilled water was used as the dissolution medium. A total of 900 mL of dissolution medium was stirred at 37 ± 0.5 °C at 50 rpm or 100 rpm. The judgment criteria I to III indicated in the “Guidelines for Bioequivalence Studies of Generic Drugs (February 29, 2012, Pharmaceutical and Food Safety Bureau Notification No. 10)” were shown in the following paragraph [19]. The compounded and stored powders were transferred to an elution container with wax paper, and a dissolution test was conducted in 12 vessels. At each vessel, 11 sampling points (0, 5, 10, 15, 30, 45, 60, 90, 120, 240, and 360 min after immersion) were taken from the dissolution test apparatus, and each sample was analyzed. At each sampling time, 1.5 mL of the eluate was collected, filtered using a 0.22 μm syringe filter (Merck KGaA, Darmstadt, Germany), and stored at −20 °C in a test vial (Waters Corporation, Tokyo, Japan) until analysis. The mean dissolution rate of each sample was compared with the compounded powders prepared on day 0 (standard preparation) to evaluate the consistency of dissolution behavior, or evaluated using the f2 function statistic [19,20].

- [Judgment Criteria I: When the standard preparation elutes a mean of 85% within 15 min]

The test preparation elutes a mean of 85% or more within 15 min. Alternatively, at 15 min, the mean dissolution rate of the test preparation is within ±15% of the mean dissolution rate of the standard preparation.

- [Judgment Criteria II: When the standard preparation elutes 85% or more within the specified test time after 30 min]

At two appropriate time points, one at 40% or 60% and the other at around 85% of the mean dissolution rate of the standard preparation, the mean dissolution rate of the test preparation is within ±15% of the mean dissolution rate of the standard preparation. Alternatively, the value of the f2 function is 45 or more.

- [Judgment Criteria III: When the mean dissolution rate of the standard preparation does not reach 85% within the specified test time]

At an appropriate time when the mean dissolution rate of the standard preparation is half the mean dissolution rate at the specified test time, and at the specified test time, the mean dissolution rate of the test preparation is within the range of the mean dissolution rate of the standard preparation ± a%. The value of a is 15 when the dissolution rate is 50% or more and 8 when the dissolution rate is less than 50%. The f2 function value is 50 or more when the dissolution rate is 50% or more, and 55 or more when the dissolution rate is less than 50%.

The f2 function (similarity factor) was used as one of the indicators for determining the similarity of dissolution behavior between formulations in dissolution tests and is represented by the following formula.

$$f2=50\;\log\left\{{\left[1+\left(\frac{1}{n}\right)\sum_{i=1}^{n}{\left(Ti-Ri\right)}^{2}\right]}^{-0.5}100\right\}$$

*Ti* and *Ri* are the mean dissolution rates of the test and standard preparations, respectively, and *n* is the number of measurement time points to be compared.

## 3. Results

### 3.1. Selection of Target Drugs Using the P-MICS Database

Using the P-MICS database, which collects prescription data from 11 hospitals and 32 clinics constructed by the National Center for Child Health and Development, we counted the number of all oral drug prescriptions for children, compounded prescriptions, and the number of facilities that carried out compounding. Overall, there were 136 compounded drug products. Of these, 24 drugs had dosage-adjustable formulations (powders or liquids). Of the 112 drugs, excluding 24 drugs, 18 were cardiovascular drugs (ambrisentan, amiodarone hydrochloride, amlodipine besylate, atenolol, bisoprolol fumarate, candesartan cilexetil, carvedilol, hydrochlorothiazide, lisinopril hydrate, losartan potassium, macitentan, pimobendane, propranolol hydrochloride, sodium beraprost, sotalol hydrochloride, tadalafil, torasemide, and verapamil hydrochloride), and the proportion of these compounded prescriptions (all of which were tablet-crushing) was 30.7% of all prescriptions. Table 1 shows the generic names of the top 50 compounded drugs in 11 hospitals (based on the number of compounded prescriptions). The cardiovascular drug with the highest number of compounded prescriptions out of all prescriptions was carvedilol (11,468 out of all 431,284 prescriptions). The drug with the highest compounded rate was hydrochlorothiazide (5682 out of 6080 hydrochlorothiazide prescriptions, 93.5%). Finally, the selected drugs for quality assessment of the compounding were carvedilol, tadalafil, hydrochlorothiazide, propranolol hydrochloride, and amlodipine besylate (ranked second, third, fourth, sixth, and seventth in the total number of compounded prescriptions, and the compounding rate for each drug was 73.1%, 83.2%, 93.5%, 72.5%, and 14.9%, respectively).

### 3.2. Detection and Quantification of Target Drugs and Their Impurities

The information on the mobile phase and separation mode in the references for each compound or impurity is shown in Table 2.

After examination using standard materials, detection methods using LC-DAD were established, and separation time, detection wavelength, retention time, and the results of calibration and quantification ranges are shown in Table 3(A). The results of the examination of LC-DAD methods validation, such as precision and accuracy of quantification for each target compound and quantifiable impurities, are shown in Table 3(B). The typical calibration curves used for the quantification of each compound are shown in Supplementary Figure S1 (calibration curve) and Table S1 (coefficient of determination, slope, and intercept). The calibration curve samples were obtained from three samples (n = 3) of different dilution series.

The separation times for the detection of amlodipine, carvedilol, hydrochlorothiazide, propranolol, and tadalafil were 25 min, 10 min, 5 min, 60 min, and 6 min, respectively, and the detection wavelengths were 250 nm, 280 nm, 273 nm, 225 nm, and 285 nm, respectively. The retention times for each compound were 7.5 min, 7.0 min, 4.2 min, 20.0 min, and 4.9 min for amlodipine, carvedilol, hydrochlorothiazide, propranolol, and tadalafil, respectively. As a result of examining the impurities (target drug), the retention times for carvedilol impurity C (carvedilol), chlorothiazide (hydrochlorothiazide), salamide (hydrochlorothiazide), propranolol impurities A, B, and C (propranolol) were 5.1 min, 3.6 min, 3.0 min, 11.5 min, 38.6 min and 50.3 min, respectively. The relative detection positions of the main components relative to the total separation time were roughly in line with previous studies [11,12,13,14,15,16]. In addition, the calibration curve for the target drug was found to be linear in the concentration range of 0.1 to 100.0 μg/mL. The LLOQ for all ingredients was 0.1 μg/mL, as determined from the S/N ratio of the calibration curve (S/N ratio > 10.0). The intra- and inter-day precision and accuracy for the QC samples were less than 15.0%.

### 3.3. Stability Testing of the Target Drugs

The results of the stability test are shown in Table 4.

When stored at 25 ± 2 °C/60 ± 5% RH, the content of all five tested drugs (amlodipine, carvedilol, hydrochlorothiazide, propranolol, and tadalafil) remained within the specification range of 90.0 to 110.0% of the initial concentration for 120 days under the three storage conditions of “bottle (closed)”, “bottle (in-use)”, and “laminated”, respectively.

### 3.4. Detection of Impurities of the Target Drugs

Table 5 shows the studied compounds, the availability of standard materials, the retention times detected or referenced, and the detection status of impurity-derived peaks in samples stored for 120 days under each storage condition.

No unknown or known impurities were detected in any of the five target drugs under any of the storage conditions.

### 3.5. Dissolution Test of the Target Drugs

The similarity of the dissolution of each drug was evaluated compared to the day 0 sample. Table 6 shows the results of the dissolution behavior similarity assessment for the five target drugs under each dissolution condition after 120 days of storage under each condition. The similarity was compared between the mean dissolution rates of samples taken at day 0, which were examined using 12 vessels, and the mean dissolution rates at each sampling point. The selected judgment criteria I to III for each test solution were also indicated. Although the dissolution behavior differs depending on the drug and the dissolution solvent, the similarity of the dissolution of the day 0 standard sample for a certain period of time was confirmed.

## 4. Discussion

The present study examined the stability, dissolution behavior, and detection of impurities of the cardiovascular drugs amlodipine, carvedilol, hydrochlorothiazide, propranolol, and tadalafil. These drugs are frequently compounded (crushed) and dispensed as powders in pediatric drug therapy. This study examined their stability after crushing the tablets and diluting them with lactose hydrate. The compounded formulation was stored under three storage conditions (bottle (closed), bottle (in-use), and laminated), assuming the storage conditions of the drug in clinical practice in Japan. The ingredient stability test, conducted over a storage period of at least 0 to 120 days, under light shielding, at 25 ± 2 °C/60 ± 5% RH, revealed that the active ingredient content was retained at a minimum of 90% during storage. No impurities were detected. In the dissolution test, no significant alterations were observed in comparison to day 0 following the compounding process, thereby indicating that storage in an amber polycarbonate container and in a cellophane polyethylene sachet after crushing and dilution with lactose hydrate is deemed acceptable in clinical practice.

In Japan, pharmaceutical companies are now beginning to provide information on the stability of the ingredients of tablets that have been crushed (not diluted with lactose or other substances) and stored under certain conditions. The products tested in this study had different compositions of excipients depending on the manufacturing and distribution company. Therefore, it is thought that the stability after crushing will differ depending on the product, but among the five components of cardiovascular drugs examined in this study, there are products that originally contain lactose as an excipient for carvedilol, hydrochlorothiazide, and tadalafil. Of these three products, the stability of carvedilol has been confirmed for 30 days at a temperature of 12.4 to 26.8 °C and 19 to 88% RH, in a room with an illuminance of around 600 lx (totaling more than 1.2 million lx·hr after 3 months), and stored in a plastic Petri dish covered with polyethylene wrap [21]. Hydrochlorothiazide was stored in a plastic Petri dish covered with polyethylene wrap in a room with a temperature of 12.7 to 26.8 °C and 24 to 88% RH, and the illumination was adjusted to approximately 600 lx (accumulating a total of more than 1.2 million lx·hr after 3 months), and its stability was confirmed for 90 days [22]. Tadalafil was stored in an open plastic Petri dish covered with polyethylene wrap under the following conditions: 25 °C, 60% RH, and 1000 lx (total cumulative illuminance of at least 400,000 lx·hr after 1 month, and at least 1.2 million lx·hr after 3 months) (once the total cumulative illuminance was reached, the Petri dish was covered with aluminum foil), and the component stability was shown for 90 days [23,24]. Regarding these three ingredients, the stability of each ingredient in the presence of lactose can be considered reasonable. On the other hand, the stability of products containing lactose as an excipient, including generic drugs, has not been demonstrated for amlodipine and propranolol. D-mannitol, or microcrystalline cellulose, is used as the main excipient for amlodipine [25,26,27]. With regard to stability after crushing, the stability of the ingredients has been reported to be at least 30 days after storing the crushed tablets under room light at a temperature of 19 to 24 °C, 50 to 92% RH, and an illuminance of 790 to 800 lx. In addition, for products that use D-mannitol as an excipient, propranolol has been shown to be stable for at least 60 days after crushing and storing in a room at 25 °C and 75% RH with light shielding [28]. Products of propranolol that use lactose as an excipient are sold, but the stability of the product after crushing has not been demonstrated by the manufacturing company [29]. In addition, the stability of the crushed tablets of all five ingredients, diluted to a certain concentration with lactose, has not been demonstrated. The fact that the stability of the product after dilution with lactose has been demonstrated in the preparation process is considered worthwhile. One of the issues is the impact of differences in the excipients contained in the products on their stability. The products tested in this study have several generic drugs, and the composition of the excipients differs depending on the manufacturing and marketing company. Therefore, it is necessary to examine each product individually, but since generic drugs are marketed by many pharmaceutical companies, studying all products may be impossible.

This study did not investigate the change of the following physical properties after compounding: particle size distribution, differential scanning calorimetry, or powder X-ray diffraction analysis. The density analysis also could not be carried out due to insufficient measurement equipment. Regarding particle size, the powder was sieved using a 500 μm mesh during the crushing process. The particle size of the crushed powder may vary depending on the crushing conditions, and this may affect solubility and dissolution characteristics. Investigating the effects of crushing conditions (rotation speed and crushing time) on particle size and solubility is considered to be a future issue. Another limitation is that the tests were only conducted under normal medical conditions (relatively mild environment). In fact, in examinations carried out in severe conditions by pharmaceutical companies, it has been reported that unpackaged or crushed products do not meet content specifications or purity requirements [21,22,23,24,25,26,27,28,29], become discolored, or increase moisture content when stored under more severe conditions. Investigating the impact of various severe conditions on the physical properties of crushed tablets compounded with lactose is considered extremely important as a future examination.

## 5. Conclusions

This study examined the pharmaceutical quality of tablets containing amlodipine, carvedilol, hydrochlorothiazide, propranolol, and tadalafil, which are commonly administered to pediatric patients following crushing and dilution with lactose. The results demonstrated that the prepared formulations were stable under light shielding at 25 ± 2 °C/60 ± 5% RH, with no impurities detected and no significant differences in dissolution behavior. Standardizing the compounding method employed in this study is expected to provide safe cardiovascular drug therapy for pediatric patients.

## Figures and Tables

**Table 1:** Pediatric compounding status in Japan based on the P-MICS database.

Rank	Genetic Name	Number of Prescriptions			Compounding Rate for Each Drug	Proportion of Compounding in All Compounded Prescriptions (/132,519)	Proportion of Compounding in All Prescriptions (/431,284)	Number of Hospitals
		Total	Compounded	Uncompounded				
1	Ramelteon	34,669	17,888	16,781	51.6%	13.50%	4.15%	11
2	Carvedilol	15,678	11,468	4210	73.1%	10.00%	2.89%	10
3	Tadalafil	10,290	8563	1727	83.2%	8.30%	2.25%	10
4	Hydrochlorothiazide	6080	5682	398	93.5%	5.98%	1.52%	9
5	Dantrolene sodium hydrate	7576	5169	2407	68.2%	5.76%	1.39%	10
6	Propranolol hydrochloride	6836	4955	1881	72.5%	5.83%	1.35%	10
7	Amlodipine besylate	33,107	4921	28,186	14.9%	6.13%	1.36%	11
8	Macitentan	5266	4645	621	88.2%	6.13%	1.40%	9
9	Azathioprine	8369	4199	4170	50.2%	5.88%	1.25%	10
10	Fluconazole	10,324	3843	6481	37.2%	5.69%	1.16%	8
11	Lisinopril hydrate	6599	3419	3180	51.8%	5.34%	1.07%	9
12	Febuxostat	12,925	3384	9541	26.2%	5.56%	1.08%	9
13	Fludrocortisone acetate	3814	2981	833	78.2%	5.16%	0.98%	10
14	Methotrexate	10,119	2959	7160	29.2%	5.37%	0.99%	9
15	Candesartan cilexetil	11,260	2900	8360	25.8%	5.54%	1.00%	10
16	Oxybutynin hydrochloride	5517	2671	2846	48.4%	5.38%	0.95%	9
17	Dexamethasone	3325	2583	742	77.7%	5.48%	0.94%	7
18	Pimobendan	2666	2300	366	86.3%	5.14%	0.84%	9
19	Ambrisentan	2657	2083	574	78.4%	4.88%	0.76%	9
20	Folic acid	6826	1994	4832	29.2%	4.89%	0.70%	9
21	Bisoprolol fumarate	3882	1986	1896	51.2%	5.10%	0.68%	10
22	Glycyrrhizinate and DL-methionine combination drug	5465	1676	3789	30.7%	4.52%	0.58%	7
23	Diazoxide	1713	1371	342	80.0%	3.86%	0.48%	10
24	Suvorexant	8917	1324	7593	14.8%	3.86%	0.46%	9
25	Fluvoxamine maleate	6982	1321	5661	18.9%	3.98%	0.47%	8
26	Sotalol hydrochloride	1595	1253	342	78.6%	3.92%	0.46%	9
27	Quetiapine fumarate	9796	1209	8587	12.3%	3.92%	0.45%	8
28	Clopidogrel sulfate	3409	1057	2352	31.0%	3.55%	0.40%	7
29	Torasemide	1674	1045	629	62.4%	3.62%	0.40%	6
30	Brotizolam	10,160	1025	9135	10.1%	3.67%	0.40%	9
31	Prazosin hydrochloride	1575	1008	567	64.0%	3.74%	0.41%	4
32	Methylprednisolone	5232	1007	4225	19.2%	3.86%	0.40%	8
33	Hydroxychloroquine sulfate	4424	979	3445	22.1%	3.89%	0.40%	8
34	Beraprost sodium	1560	796	764	51.0%	3.28%	0.33%	8
35	Trazodone hydrochloride	2517	731	1786	29.0%	3.10%	0.30%	7
36	Amiodarone hydrochloride	1048	704	344	67.2%	3.07%	0.29%	10
37	Thiamazole	7924	695	7229	8.8%	3.11%	0.29%	10
38	Colchicine	1881	684	1197	36.4%	3.15%	0.29%	9
39	Olanzapine	6454	630	5824	9.8%	2.98%	0.27%	8
40	Metronidazole	1280	590	690	46.1%	2.86%	0.26%	10
41	Lanthanum carbonate hydrate	857	589	268	68.7%	2.93%	0.26%	3
42	Rufinamide	2377	563	1814	23.7%	2.87%	0.25%	9
43	Vonoprazan fumarate	3598	541	3057	15.0%	2.83%	0.24%	6
44	Pyridoxal phosphate hydrate	2119	467	1652	22.0%	2.51%	0.21%	7
45	Rifampicin	574	456	118	79.4%	2.50%	0.20%	7
46	Sertraline hydrochloride	6883	455	6428	6.6%	2.55%	0.20%	9
47	Sulthiame	1703	429	1274	25.2%	2.46%	0.20%	8
48	Losartan potassium	2808	423	2385	15.1%	2.48%	0.19%	9
49	Cobamamide	672	421	251	62.6%	2.52%	0.19%	4
50	Conjugated estrogen	1961	407	1554	20.8%	2.49%	0.09%	9
Total of rankings 51st and below		116,341	8070	108,271	6.9%	50.61%	1.87%	

Only the top 50 most prescribed crushed drug formulas are listed.

**Table 2:** Mobile phase and separation mode of liquid chromatography.

Target Formulation	Target Compound	Mobile Phase A	Mobile Phase B	Separation Mode [Time (min)/% Mobile Phase B]
Amlodipine besylate	Amlodipine	20 mM sodium phosphate buffer solution (adjusted to pH 3.4)	acetonitrile and water (90:10, *v*/*v*)	Linear gradient [0/15, 10/55, 12/80, 25/80, 25/15, 25/15]
Carvedilol	Carvedilol	water: acetonitrile (45:55, *v*/*v*)		Isocratic [0/45, 10/45]
	Carvedilol impurity C	water: acetonitrile (45:55, *v*/*v*)		Isocratic [0/45, 10/45]
Hydrochlorothiazide	Hydrochlorothiazide	phosphoric acid solution: acetonitrile (90: 10, *v*/*v*) adjusted to pH 3.6 with diluted phosphoric acid		Isocratic [0/90, 5/90]
	Chlorothiazide	phosphoric acid solution: acetonitrile (90: 10, *v*/*v*) adjusted to pH 3.6 with diluted phosphoric acid		Isocratic [0/90, 5/90]
	Salamide	phosphoric acid solution: acetonitrile (90: 10, *v*/*v*) adjusted to pH 3.6 with diluted phosphoric acid		Isocratic [0/90, 5/90]
Propranolol hydrochloride	Propranolol	15 mM ammonium phosphate solution (adjusted to pH 2.3)	Methanol: acetonitrile: isopropyl alcohol (24:50:26, *v*/*v*/*v*)	Linear gradient [0/20, 30/46, 45/65, 45/20, 60/20]
	Propranolol impurity A	15 mM ammonium phosphate solution (adjusted to pH 2.3)	Methanol: acetonitrile: isopropyl alcohol (24:50:26, *v*/*v*/*v*)	Linear gradient [0/20, 30/46, 45/65, 45/20, 60/20]
	Propranolol impurity B	15 mM ammonium phosphate solution (adjusted to pH 2.3)	Methanol: acetonitrile: isopropyl alcohol (24:50:26, *v*/*v*/*v*)	Linear gradient [0/20, 30/46, 45/65, 45/20, 60/20]
	Propranolol impurity C	15 mM ammonium phosphate solution (adjusted to pH 2.3)	Methanol: acetonitrile: isopropyl alcohol (24:50:26, *v*/*v*/*v*)	Linear gradient [0/20, 30/46, 45/65, 45/20, 60/20]
Tadalafil	Tadalafil	0.1% formic acid solution	Acetonitrile	Linear gradient [0/73, 2/65, 2.5/65, 4/32, 5/32, 6/63]

**Table 3:** (**A**). Detection and calibration range. (**B**). Accuracy and precision assessment.

(A) Detection and Calibration Range													
Target Formulation	Target Compound	Separation Time		Detection Wavelength		Retention Time		Calibration Range (µg/mL)		LLOQ (µg/mL)		ULOQ (µg/mL)	
Amlodipine besylate	Amlodipine	25.0 min		250 nm		7.5 min		0.1–100.0		0.1		100.0	
Carvedilol	Carvedilol	10.0 min		280 nm		7.0 min		0.1–100.0		0.1		100.0	
	Carvedilol impurity C	10.0 min		280 nm		5.1 min		0.1–100.0		0.1		100.0	
Hydrochlorothiazide	Hydrochlorothiazide	5.0 min		273 nm		4.2 min		0.1–100.0		0.1		100.0	
	Chlorothiazide	5.0 min		273 nm		3.6 min		0.1–100.0		0.1		100.0	
	Salamide	5.0 min		273 nm		3.0 min		0.1–100.0		0.1		100.0	
Propranolol hydrochloride	Propranolol	60.0 min		225 nm		20.0 min		0.1–100.0		0.1		100.0	
	Propranolol impurity A	60.0 min		225 nm		11.5 min		0.1–100.0		0.1		100.0	
	Propranolol impurity B	60.0 min		225 nm		38.6 min		0.1–100.0		0.1		100.0	
	Propranolol impurity C	60.0 min		225 nm		50.3 min		0.1–100.0		0.1		100.0	
Tadalafil	Tadalafil	6.0 min		285 nm		4.9 min		0.1–100.0		0.1		100.0	
**(B) Accuracy and Precision Assessment**													
**Target Formulation**	**Target Compound**	**Interday**						**Intraday**					
		**Accuracy**			**Precision**			**Accuracy**			**Precision**		
		**LQC**	**MQC**	**HQC**	**LQC**	**MQC**	**HQC**	**LQC**	**MQC**	**HQC**	**LQC**	**MQC**	**HQC**
Amlodipine besylate	Amlodipine	13.2 ±1.9%	4.8 ±1.4%	6.0 ±1.9%	2.8 ±4.9%	15.0 ±1.0%	15.0 ±3.9%	5.7 ±6.9%	2.7 ±4.9%	14.3 ±4.1%	9.7 ±4.0%	9.8 ±0.2%	9.0 ±4.2%
Carvedilol	Carvedilol	10.9 ±1.7%	7.0 ±1.7%	12.0 ±1.9%	7.7 ±1.4%	7.9 ±1.9%	10.9 ±3.9%	12.5 ±3.0%	11.0 ±1.9%	14.3 ±5.3%	8.8 ±5.3%	8.8 ±5.3%	6.5 ±5.3%
	Carvedilol impurity C	11.2 ±2.9%	5.2 ±2.1%	3.6 ±1.9%	2.7 ±3.9%	7.9 ±2.3%	7.8 ±3.9%	12.0 ±2.0%	8.3 ±1.7%	9.9 ±3.2%	10.6 ±5.6%	11.8 ±4.2%	13.7 ±3.2%
Hydrochlorothiazide	Hydrochlorothiazide	9.6 ±3.6%	4.7 ±3.6%	8.4 ±3.6%	10.6 ±2.9%	3.4 ±0.9%	9.3 ±1.9%	8.1 ±4.7%	10.7 ±1.4%	6.8 ±4.5%	13.3 ±3.2%	10.8 ±4.2%	5.2 ±1.1%
	Chlorothiazide	5.5 ±1.6%	2.8 ±3.9%	5.5 ±1.9%	7.8 ±1.4%	12.7 ±1.4%	14.6 ±3.4%	11.2 ±3.4%	5.3 ±1.8%	7.5 ±5.2%	3.9 ±3.2%	12.0 ±4.2%	13.8 ±1.9%
	Salamide	10.7 ±1.3%	13.2 ±1.4%	11.4 ±1.9%	7.8 ±5.4%	4.3 ±1.4%	7.3 ±4.4%	11.0 ±1.8%	1.4 ±3.4%	6.6 ±3.2%	7.6 ±4.8%	14.1 ±4.2%	14.7 ±4.6%
Propranolol hydrochloride	Propranolol	12.7 ±1.9%	11.3 ±1.3%	11.2 ±3.9%	3.6 ±3.3%	3.3 ±3.3%	4.1 ±1.7%	9.7 ±1.9%	5.9 ±0.6%	5.6 ±4.7%	10.6 ±4.6%	2.8 ±4.2%	7.0 ±3.0%
	Propranolol impurity A	5.9 ±3.0%	8.4 ±1.5%	14.5 ±2.9%	4.8 ±1.8%	10.0 ±5.5%	13.6 ±1.9%	11.0 ±4.4%	3.3 ±2.4%	9.1 ±5.0%	10.4 ±4.2%	9.7 ±4.4%	12.0 ±5.0%
	Propranolol impurity B	11.6 ±2.9%	15.0 ±3.9%	11.6 ±1.9%	10.8 ±4.4%	5.5 ±1.4%	9.3 ±1.4%	12.7 ±3.3%	12.4 ±1.7%	13.3 ±4.0%	11.2 ±4.2%	14.9 ±2.7%	6.4 ±4.2%
	Propranolol impurity C	14.3 ±5.2%	12.5 ±4.4%	4.1 ±3.2%	7.6 ±4.0%	6.9 ±4.2%	5.4 ±4.6%	12.0 ±4.4%	15.0 ±3.3%	4.4 ±4.0%	12.1 ±4.2%	8.2 ±1.8%	8.1 ±4.0%
Tadalafil	Tadalafil	11.0 ±4.7%	5.6 ±4.7%	3.3 ±3.2%	8.7 ±3.5%	10.8 ±4.2%	9.9 ±4.2%	4.4 ±4.2%	4.0 ±1.1%	9.4 ±3.2%	13.4 ±1.2%	3.4 ±5.2%	3.7 ±3.1%

LLOQ, the lower limit of quantification; ULOQ, the upper limit of quantification; 0.2, 2.0, and 20.0 µg/mL spiked standard materials were used for low-, middle-, and high-quality control samples (LQC, MQC, and HQC), respectively. Quality control samples were prepared in triplicate (n = 3), and the means with standard deviation were presented.

**Table 4:** Mean contents of target ingredients after compounding and storage in each condition.

Target Compound	Mean Relative Contents (n = 3)														
	Storage Condition I: Bottle (Closed)					Storage Condition II: Bottle (In-Use)					Storage Condition III: Laminated				
	Day 0	Day 30	Day 60	Day 90	Day 120	Day 0	Day 30	Day 60	Day 90	Day 120	Day 0	Day 30	Day 60	Day 90	Day 120
Amlodipine	100.0	102.5 ±3.2%	99.8 ±3.1%	94.7 ±2.5%	96.6 ±5.2%	100.0	95.8 ±1.6%	107.7 ±6.3%	99.2 ±3.3%	99.2 ±2.6%	100.0	107.4 ±1.6%	104.6 ±1.9%	96.3 ±4.6%	102.3 ±2.1%
Carvedilol	100.0	104.7 ±4.4%	96.5 ±2.5%	103.9 ±3.9%	97.8 ±4.9%	100.0	103.5 ±2.3%	97.7 ±3.6%	97.3 ±6.3%	100.8 ±1.3%	100.0	103.7 ±5.2%	98.9 ±4.2%	103.8 ±4.5%	105.8 ±2.1%
Hydrochlorothiazide	100.0	98.2 ±1.7%	96.1 ±1.9%	100.5 ±3.1%	102.5 ±2.6%	100.0	98.7 ±3.8%	107.9 ±3.9%	99.1 ±3.2%	98.4 ±2.8%	100.0	96.2 ±5.8%	104.2 ±6.2%	102.1 ±1.1%	96.0 ±3.4%
Propranolol	100.0	101.3 ±5.5%	104.7 ±6.1%	98.4 ±2.0%	100.9 ±3.3%	100.0	102.9 ±1.7%	103.0 ±3.4%	94.7 ±2.0%	97.3 ±2.8%	100.0	106.7 ±4.7%	97.5 ±5.1%	95.0 ±2.6%	94.8 ±4.4%
Tadalafil	100.0	97.3 ±0.9%	102.4 ±1.8%	99.4 ±4.2%	97.5 ±3.0%	100.0	108.5 ±2.5%	99.1 ±2.5%	99.3 ±3.5%	96.0 ±5.2%	100.0	97.3 ±3.7%	103.3 ±3.9%	102.2 ±2.9%	94.9 ±2.8%

The relative contents after storage in each storage condition were calculated. The mean values with standard deviations of three prepared samples were indicated.

**Table 5:** Impurity detection status after compounding.

Target Formulation	Target Compound	Type	Availability of Standard Materials	Reference Retention Time ^a^	Mean Relative Peak Area ^b^		
					Storage Condition I: Bottle (Closed)	Storage Condition II: Bottle (In-Use)	Storage Condition III: Laminated
Amlodipine besylate	Amlodipine	Target compound	Available	7.5 min	100.0%	100.0%	100.0%
	Amlodipine impurity A	Impurity	None	> 20.0 min	No detection	No detection	No detection
	Amlodipine impurity B	Impurity	None	15.0–20.0 min	No detection	No detection	No detection
	Amlodipine impurity D	Impurity	None	0.0–5.0 min	No detection	No detection	No detection
	Amlodipine impurity E	Impurity	None	5.0–10.0 min	No detection	No detection	No detection
	Amlodipine impurity F	Impurity	None	0.0–5.0 min	No detection	No detection	No detection
	Amlodipine impurity G	Impurity	None	10.0–15.0 min	No detection	No detection	No detection
	Amlodipine impurity H	Impurity	None	15.0–20.0 min	No detection	No detection	No detection
Carvedilol	Carvedilol	Target compound	Available	7.0 min	100.0%	100.0%	100.0%
	Carvedilol impurity C	Impurity	Available	5.1 min	<0.01%	<0.01%	<0.01%
Hydrochlorothiazide	Hydrochlorothiazide	Target compound	Available	4.2 min	100.0%	100.0%	100.0%
	Chlorothiazide	Impurity	Available	3.6 min	<0.01%	<0.01%	<0.01%
	Salamide	Impurity	Available	3.0 min	No detection	No detection	No detection
Propranolol hydrochloride	Propranolol	Target compound	Available	20.0 min	100.0%	100.0%	100.0%
	Propranolol impurity A	Impurity	Available	11.5 min	No detection	No detection	No detection
	Propranolol impurity B	Impurity	Available	38.6 min	<0.01%	<0.01%	<0.01%
	Propranolol impurity C	Impurity	Available	50.3 min	No detection	No detection	No detection
Tadalafil	Tadalafil	Target compound	Available	4.0 min	100.0%	100.0%	100.0%
	Tadalafil impurity A	Impurity	None	1.0–2.0 min	No detection	No detection	No detection
	Tadalafil impurity B	Impurity	None	1.0–2.0 min	No detection	No detection	No detection
	Tadalafil impurity C	Impurity	None	4.0–4.2 min	<0.01%	<0.01%	<0.01%
	Tadalafil impurity D	Impurity	None	> 4.2 min	No detection	No detection	No detection
	Tadalafil impurity E	Impurity	None	> 4.2 min	No detection	No detection	No detection

^a^, The retention time of impurities for which standard products were unavailable is displayed as a reference value. ^b^, Results after 120 days of storage are shown. No detection, S/N < 1.0.

**Table 6:** Similarity of dissolution behavior after compounding for 120 days.

Storage Conditions	Test Solutions		Similarity of Each Target Compounds ^c^ (Judgement Criteria)				
			Amlodipine	Carvedilol	Hydrochlorothiazide	Propranolol	Tadalafil
Bottle (closed)	50 rpm	pH 1.2: First fluid in the Japanese Pharmacopoeia dissolution test	−7.1% (III)	−1.8% (III)	(f2 = 55.0) ^d^ (II)	7.1% (II)	−6.1% (III)
		pH 5.0: Diluted McIlvaine buffer solution	9.4% (II)	−7.8% (II)	5.3% (II)	10.0% (I)	10.0% (III)
		pH 6.8: Second fluid in the Japanese Pharmacopoeia dissolution test	−4.1% (II)	−7.5% (II)	−4.7% (II)	−1.6% (I)	9.5% (III)
		Water: Purified water in the Japanese Pharmacopoeia	−3.9% (II)	−5.9% (III)	−8.1% (III)	5.6% (I)	−3.8% (III)
	100 rpm	pH 6.8: Second fluid in the Japanese Pharmacopoeia dissolution test	−9.5% (I)	0.9% (II)	5.4% (I)	−7.9% (I)	8.4% (III)
Bottle (in-use)	50 rpm	pH 1.2: First fluid in the Japanese Pharmacopoeia dissolution test	−2.9% (III)	9.9% (III)	−8.3% (II)	0.4% (II)	−6.1% (III)
		pH 5.0: Diluted McIlvaine buffer solution	0.7% (II)	8.9% (II)	9.4% (II)	−8.1% (I)	1.6% (III)
		pH 6.8: Second fluid in the Japanese Pharmacopoeia dissolution test	−9.7% (II)	−8.3% (II)	−6.7% (II)	6.3% (I)	8.3% (III)
		Water: Purified water in the Japanese Pharmacopoeia	4.3% (II)	0.5% (III)	9.8% (III)	6.6% (I)	8.6% (III)
	100 rpm	pH 6.8: Second fluid in the Japanese Pharmacopoeia dissolution test	5.9% (I)	8.4% (II)	1.2% (I)	−8.6% (I)	10.4% (III)
Laminated	50 rpm	pH 1.2: First fluid in the Japanese Pharmacopoeia dissolution test	−8.1% (III)	7.6% (III)	3.3% (II)	−7.7% (II)	−3.0% (III)
		pH 5.0: Diluted McIlvaine buffer solution	1.1% (II)	−6.0% (II)	−8.8% (II)	9.1% (I)	0.2% (III)
		pH 6.8: Second fluid in the Japanese Pharmacopoeia dissolution test	−7.6% (II)	9.1% (II)	−6.1% (II)	−8.1% (I)	7.9% (III)
		Water: Purified water in the Japanese Pharmacopoeia	9.6% (II)	8.6% (III)	−3.5% (III)	1.8% (I)	2.0% (III)
	100 rpm	pH 6.8: Second fluid in the Japanese Pharmacopoeia dissolution test	2.7% (I)	8.6% (II)	9.9% (I)	−0.7% (I)	0.2% (III)

^c^, The similarity was compared between the mean dissolution rates of samples taken at Day 0, which were examined using 12 vessels, and the mean dissolution rates at each sampling point. ^d^, The f2 function (similarity factor) was calculated as follows: $f2=50\log\left\{{\left[1+\left(\frac{1}{n}\right)\sum_{i=1}^{n}{\left(Ti-Ri\right)}^{2}\right]}^{-0.5}100\right\}$ *Ti* and *Ri* are the mean dissolution rates of the test and standard preparations, respectively, and *n* is the number of measurement time points to be compared. The difference in mean dissolution rates between the test formulation (n = 12) and the reference formulation (on the day of compounding) was evaluated.

## Data Availability

The data presented in this study are available on request from the corresponding author.

## Supplementary Materials

The following supporting information can be downloaded at: https://www.mdpi.com/article/10.3390/pharmaceutics17040535/s1, Figure S1: Typical calibration curves for target compounds; Table S1: Coefficient of determination, slope, and intercept for the typical calibration curves.

## Abbreviations

The following abbreviations are used in this manuscript:

P-MICS	Pediatric Medical Information Collection System
LC	Liquid chromatography
DAD	Diode array detection
HQC	High-quality control
MQC	Middle-quality control
LQC	Low-quality control
LLOQ	The lower limit of quantification
ULOQ	The upper limit of quantification
S/N	Signal-to-noise
ICH	The International Council for Harmonization of Technical Requirements for Pharmaceuticals for Human Use
RH	Relative humidity
