# Supplementary material for: Stability and Dissolution Behavior Changes After Drug Compounding for Pediatric Cardiovascular Pharmacotherapy
Source: Pharmaceutics. 2025 Apr 19;17(4):535. doi: 10.3390/pharmaceutics17040535 (PMC12030496; doi:10.3390/pharmaceutics17040535)
Supplement: Supplementary file 1 [file pharmaceutics-17-00535-s001.zip › pharmaceutics-3589689-supplementary.pdf]

pharmaceutics

MDPI

Supplementary Figure S1. Typical calibration curves for target compounds. The typical calibration curves used for the detection of Amlodipine (A), Carvedilol (B), Carvedilol impurity-C (C), Hydrochlorothiazide (D), Chlorothiazide (E), Salamide (F), Propranolol (G), Propranolol impurity-A (H), Propranolol impurity-B (I), Propranolol impurity-C (J), and Tadalafil were shown. The calibration curve consists of seven points, and each point shown in the figure indicates the average of the peak area (mAU * min) obtained from three samples (n=3).

(A) Amlodipine

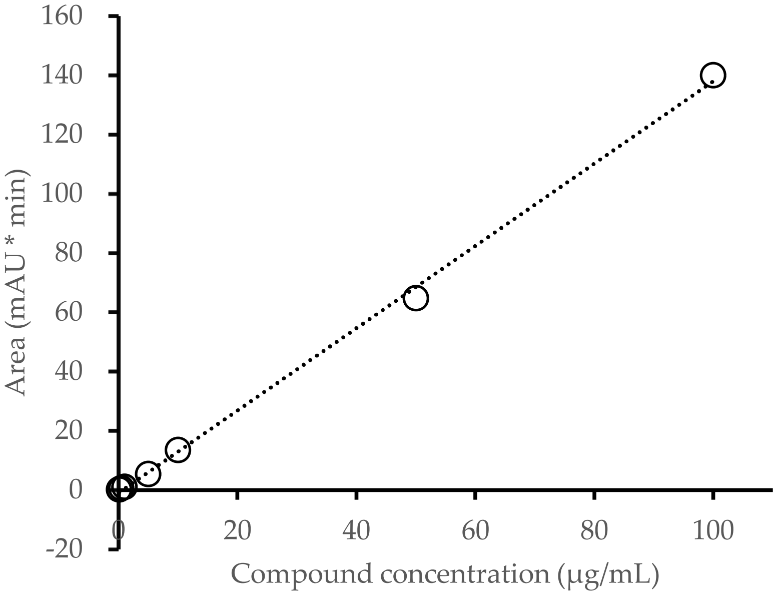

(B) Carvedilol

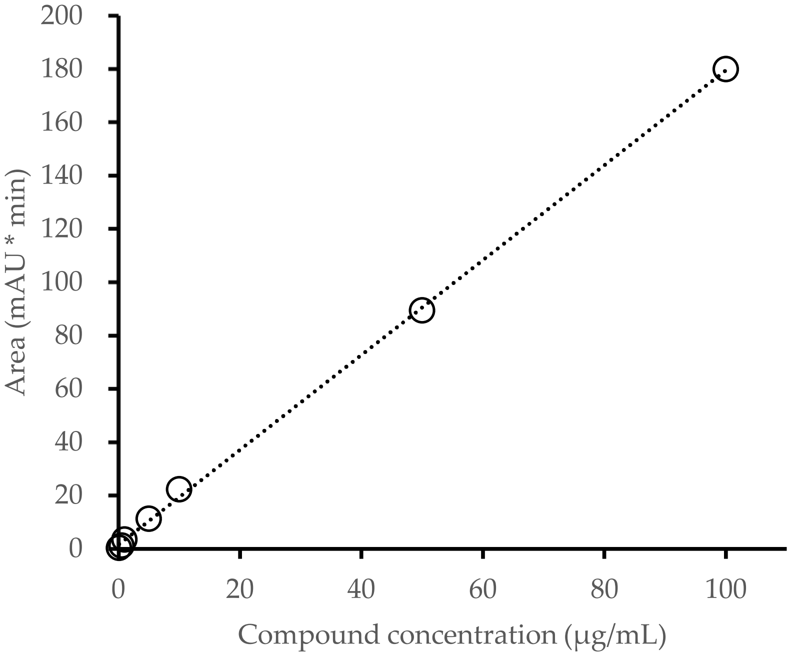

 https://doi.org/10.3390/xxxxx

(C) Carvedilol impurity-C

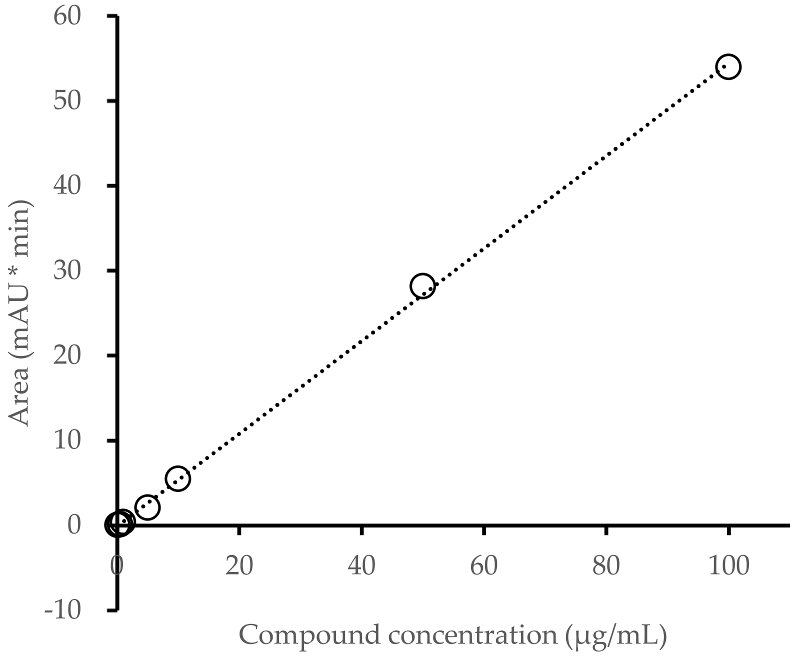

(D) Hydrochlorothiazide

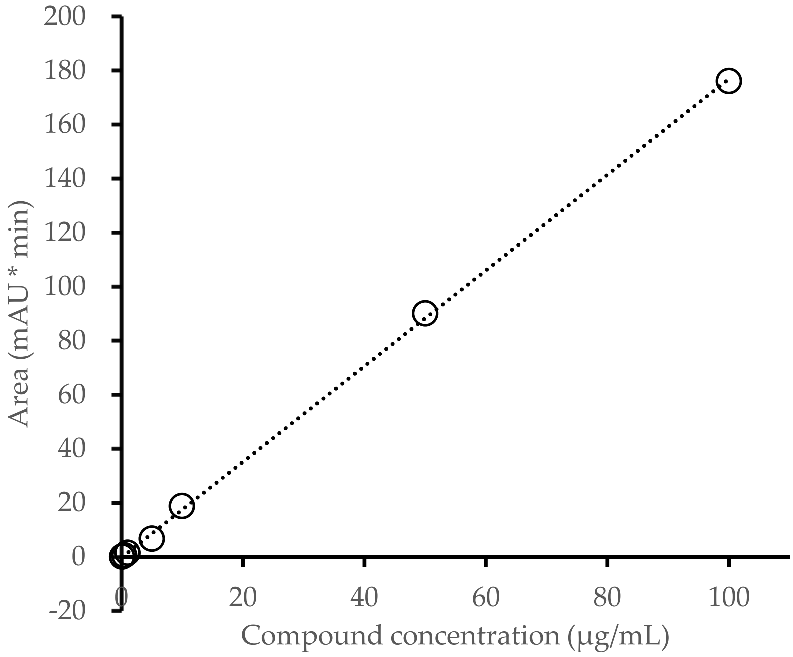

(E) Chlorothiazide

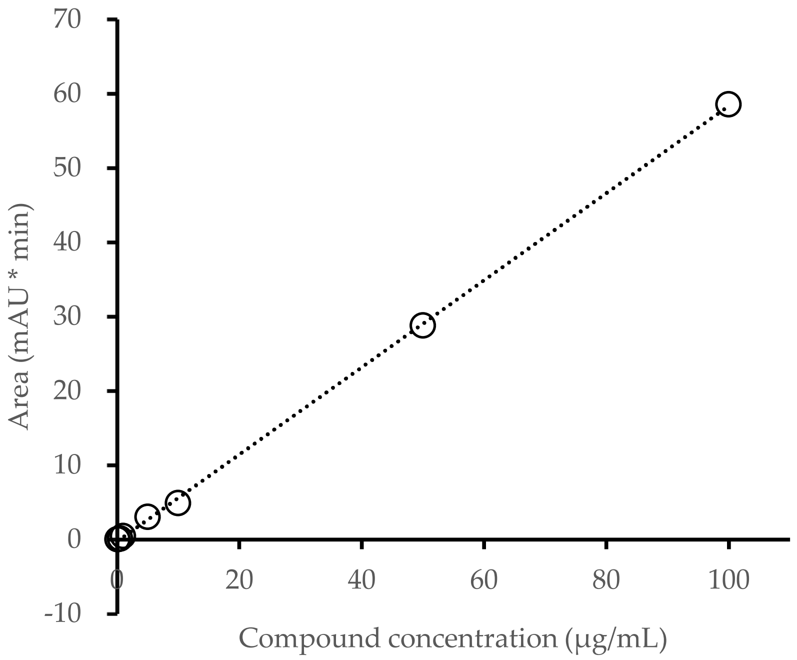

(F) Salamide

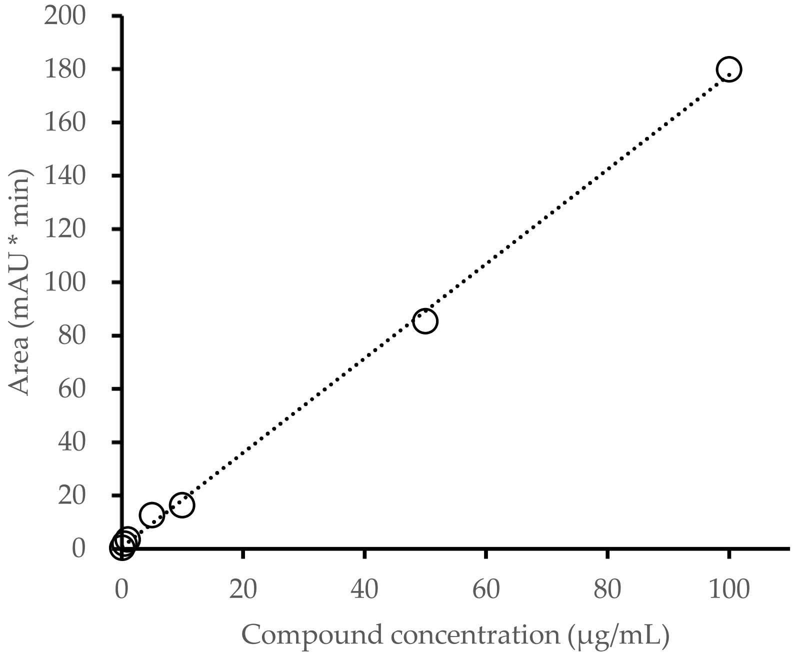

(G) Propranolol

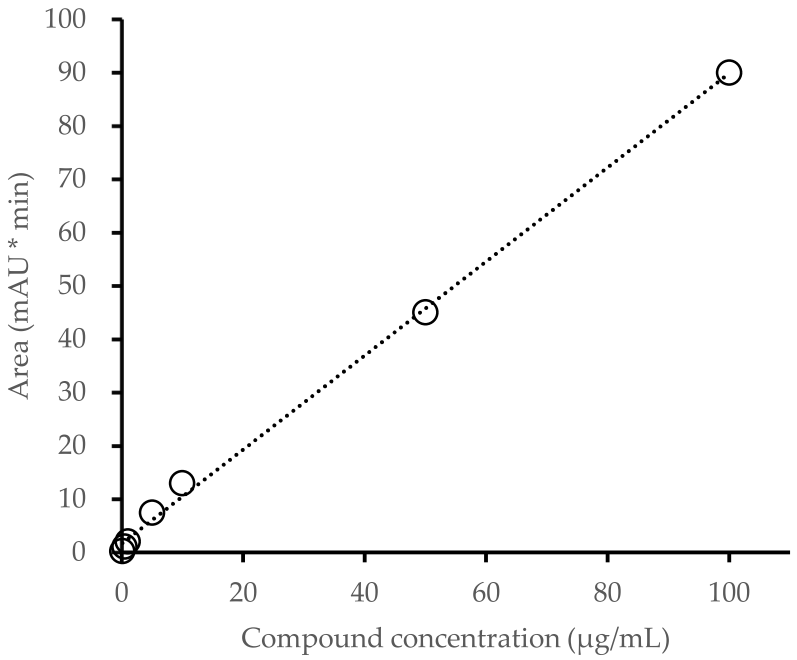

(H) Propranolol impurity-A

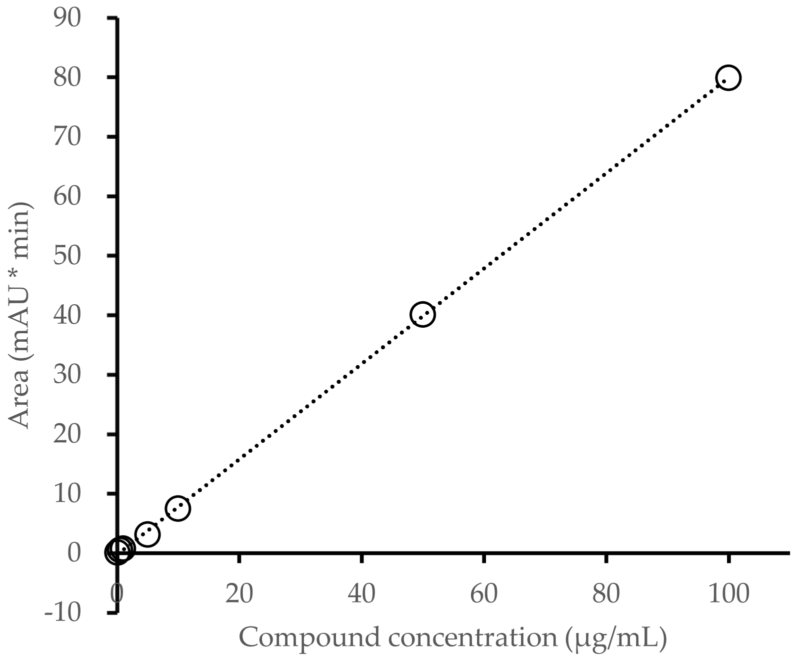

(I) Propranolol impurity-B

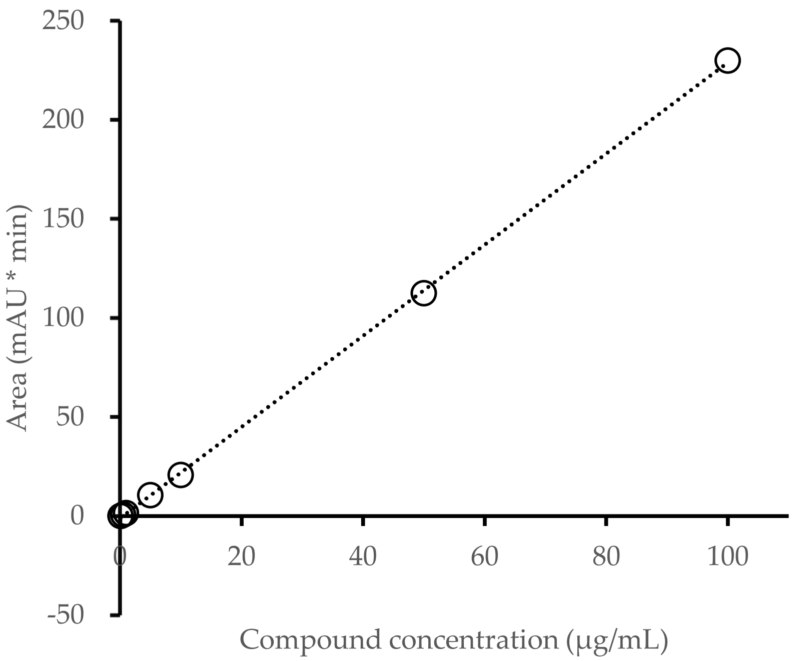

(J) Propranolol impurity-C

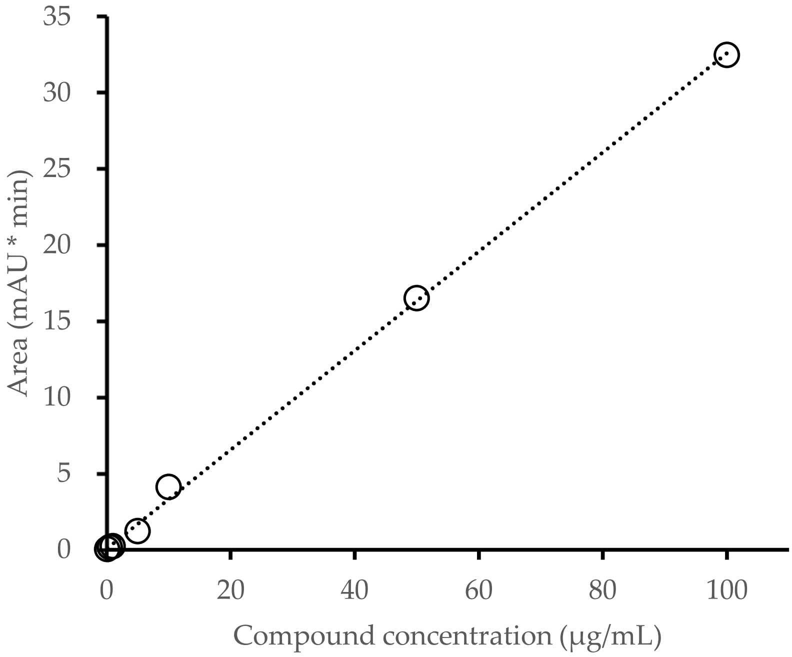

(K) Tadalafil

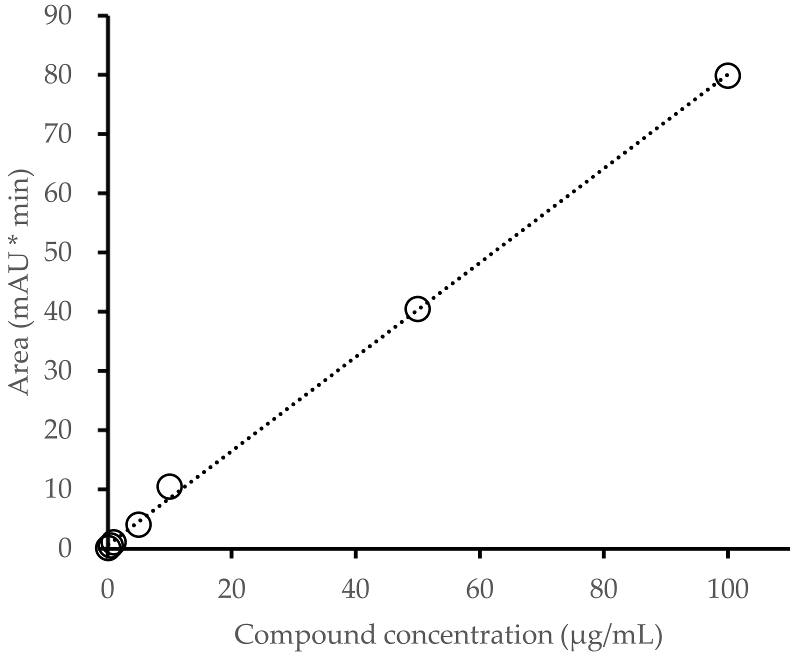

Supplementary Table S1. Coefficient of determination, slope, and intercept for the typical calibration curves. The typical calibration curve determination coefficients, slopes, and intercepts used for the target compounds were shown.

Compound names	**Amlodipine**	**Carvedilol**	**Carvedilol impurity-C**	**Hydrochlorothiazide**	**Chlorothiazide**	**Salamide**	**Propranolol**	**Propranolol impurity-A**	**Propranolol impurity-B**	**Propranolol impurity-C**	**Tadalafil**
Coefficient of determination (r^2)	0.999	0.997	0.995	0.998	0.996	0.997	0.997	0.996	0.998	0.998	0.998
Slope	1389.6	1780.6	545.4	1771.8	586.3	1772.3	882.4	802.0	2300.5	325.5	795.1
Intercept	-923.7	1587.7	-96.6	-184.7	-262.3	692.9	1668.0	-208.0	-967.7	70.9	615.4
